# Professional Role Transition in Nursing: Leveraging Transition Theory to Mitigate the Current Human Resource Crises

**DOI:** 10.3390/healthcare13060671

**Published:** 2025-03-19

**Authors:** Stella Akomeng Aryeequaye, Kathryn Corneau, Judy E. Duchscher

**Affiliations:** School of Nursing, Kamloops, Thompson Rivers University, Kamloops, BC V2C 0C8, Canada; aryeequayes23@mytru.ca (S.A.A.); kathryncorneau@gmail.com (K.C.)

**Keywords:** new graduates, transition, retention, professional development, professional role, nurses

## Abstract

New graduate nurse (NGN) turnover is emerging as one of the foremost issues in healthcare systems, primarily due to the implications for patient care and the need to secure the human resource future of the nursing profession. The initial months of transitioning into the professional role are crucial for cultivating and developing clinical practice patterns, professional values and a connection to the profession. However, the initial transition period for new nurses is associated with numerous challenges that can interrupt a healthy introduction into practice, justifying the critical prioritization of these issues. In light of these challenges to NGN entry to practice, this paper aims to conceptualize the contemporary professional role transition experiences of new graduate nurses and highlight the potential leverage that transition theories offer in managing this experience. Eleven transition theories relevant to this discourse were identified to enhance the understanding and comprehension of the new graduate nurses to inform future initiatives, directives, interventions and policies.

## 1. Introduction

Nurses are indispensable to optimizing healthcare delivery and form an integral part of the healthcare system. Increasingly, there have been concerns about a decline in the nursing workforce in healthcare systems across the globe, particularly in the aftermath of the pandemic [1,2,3,4,5,6,7,8]. In the 2020 state of the world’s nursing report, attention was drawn to an anticipated shortage in the nursing workforce [9]. According to the report, the estimated shortage of nurses by 2030 from 2018 data was 5.7 million [9]. One out of every six of the world’s nurses is also expected to retire within the next decade, indicating a need to train and employ at least 4.7 million nurses to bridge the gap that will be created by the retiring nurses alone [9] In a separate brief, the ICN identified that 20% of its National Nurses Associations had witnessed a dramatic increase in nursing turnover during the pandemic, and further projected that, globally, about 10 million nurses will be needed to fill the gap created by retiring nurses and the after-effects of COVID-19 [10]. Key deficiencies are also expected in Africa, Southeast Asia, WHO Eastern Mediterranean Region and parts of Latin America [11]. Staffing shortages in nursing are not new; rather, some have suggested that issues related to culture, workload and gender-based professional oppression have existed for decades but were exacerbated by the strains of a global pandemic [12,13,14,15].

### Background and Context

Nursing in direct care has always been fast-paced and stressful, but the fiscal limitations and human resource pressures felt by healthcare organizations over the past several decades have accelerated the negative impacts on the overall well-being of nurses [16,17,18,19,20]. Individual factors aside, the cumulative effect of deteriorating workplace conditions has been cited as the primary motivation for escalating attrition, further aggravating the ongoing shortage witnessed across healthcare systems [21,22,23,24,25,26]. While nursing associations have advocated for the training of more nursing students as a means of narrowing the shortage gaps, this approach does not address the underlying causes of attrition in early to mid-career nurses [27]. Ren et al.’s (2024) [13] meta-analysis of the global nurse turnover rate since COVID revealed an attrition rate between 8% and 36.6%. Falatah’s (2021) [28] study revealed that, although predictors of turnover intentions pre- and post-COVID differed, the post-pandemic intention to leave rates were considerably higher than pre-pandemic rates. Wu et al. (2024) [29] reported comparable turnover rates to Ren et al.’s findings (2024) [13], with the majority of those leaving being NGNs. In Canada, it has been estimated that 18–30% and 37–57% of NGNs are leaving the workplace within their first and second year of practice, respectively [30]. The growing turnover rate amongst newly graduated nurses is a canary in the coal mine moment. The Emergency Care Research Institute (ECRI) Top 10 Patient Safety Concerns for 2024 suggests that we are poised for an unprecedented healthcare crisis that threatens patient safety globally should we be unable to address issues related to professional role transition that are driving new professionals from the workplace [31].

An NGN can be described as someone who has completed their nursing education and has up to 12 months of experience as a professional nurse [32]. Typically, they have limited experience and expertise upon entering the professional role, gradually developing the competencies and confidence required for their new responsibilities over time [33]. Significant research has explored the experiences of NGNs [34,35]. While it is often a time of personal growth and fulfilment, the initial entry to practice is also marked by significant stress and unmet expectations [30,34,35]. Upon their first fully responsible and accountable entry to practice, NGNs suddenly find themselves in roles that demand increased and advanced knowledge application, acclimation to culturally nuanced practice environments and the expectation of inter- and intra-disciplinary collaboration [32,36,37]. These new and shifting roles and responsibilities can be overwhelming, particularly in the face of increasingly complex patient populations [36,37,38,39]. Studies suggest that particular aspects of the profession, such as heavy workloads, job demands, stress, sleep disturbances, workplace incivility, feelings of inadequacy, disappointment, poor interprofessional relationships and insufficient managerial and organizational support often ‘come as a surprise’ to NGNs, and strongly influence early career exit decisions [40,41,42,43,44,45]. Masso et al. (2022) [46] identified the receptivity of the clinical environment as a key element in retaining NGNs, a finding echoed by other studies that correlate supportive environments with retention [47,48,49,50,51,52,53,54]. Creating a supportive environment that allows NGNs to thrive in practice requires a deep understanding of their transition process and experiences, which can be informed by transition theories. This paper aims to describe key transition theories, with the primary goal of deepening our understanding of how NGNs navigate the process of transitioning into professional practice, thereby informing pathways to success.

## 2. Overview of Transition Theories

Theories offer frameworks that explain the relationships between various aspects of reality and are used to guide and inform practice [55,56]. Broad professional role transition theories, as well as those specific to nursing, offer structured approaches for understanding and, this paper argues, optimizing the experiences of NGNs undergoing changes in their professional status [57,58]. There are several seminal and contemporary theoretical frameworks and conceptual models that offer valuable insight into how individuals navigate and adapt to major life changes. The sum total of an NGN’s experiences during professional role transition reflects a period imbued with significant and simultaneous physical, psychosocial, developmental, cultural and spiritual influences, impacted by external factors [32]. Theories that convey the intricate nuances of the transition process have relevance for the NGN professional role transition. After a comprehensive review of relevant literature, eleven such frameworks were identified because they addressed role transition, life transition, organizational change, skill acquisition and development and new graduate nurse transition, which are relevant to the discussion. Salient theoretical overviews were written and forwarded for vetting and editing to all theorists, except for Bridges and Kramer who are deceased. This approach ensured that the theorists chosen were both well-supported by existing academic research and aligned with expert recommendations, thereby enhancing the rigour and relevance of the comparative analysis.

### 2.1. Nicholson’s Work-Role Transition Theory

The process of adapting to new work roles in organizations is addressed in Nicholson’s work-role theory [59]. An organization’s structure can shape an individual’s emotions, sense of identity and behaviour during a transition [59]. Depending on the role requirement, the individual’s motivation orientation, prior occupational socialization and the current induction socialization process, incumbents adopt an adjustment strategy [59]. Four main modes of adjustment emerge [59]. Replication and absorption adjustment modes focus on personal development, and determination and exploration focus on developing the role [59]. While some people reproduce old role behaviours in new roles (replication), others cultivate new skills and amend their behaviour to conform to new role expectations (absorption). Those practicing determination significantly change their work within the organization to align with their current skills and behaviour but maintain their identity in the new role. Exploring individuals initiate and implement personal and organizational changes concurrently in their new role [59]. Work roles that offer growth opportunities build capacity for innovation and those where insight from prior experiences remains relevant lead to personal development [59]. Socialization approaches employed in the new role determine the extent of growth experienced personally or role-wise. Cumulative learning, use of role models and divestiture foster personal development, and random learning with no role models and investiture shifts the person into role development [59]. In 1990, Nicholson and West introduced the Transition Cycle Model, which has four distinct and interrelated stages representing the role transition course: preparation; encounter; adjustment; stabilization. The encounter stage marks the feeling and sense-making phase. Expectations and realities are reconciled here, subject to adequate preparations. During the adjustment stage, strategy modes are adopted based on the work environment and the individual’s disposition. The final stage of stabilization is attained when the individual is attuned to the role and its expectations and advances toward seeking growth opportunities [60].

### 2.2. Hardy and Conway’s Role Theory

Hardy and Conway’s role theory was iterated from an interactionist perspective to illustrate how people’s actions and responses are shaped by their interpretation of the actions of others [61]. This role theory is a collection of concepts and a variety of hypothetical formulations that predict people’s behaviour in situations [61]. The unpredictable nature of human interactions means that individuals must build the capacity to respond to the unanticipated actions of others [61]. The expected behaviours of others are learnt from socializing agents, who are usually families, peers and institutions. These agents transmit the requisite social learning. Then, over time and through interactional and observational learning, the individual, now a new professional, enacts their role in accordance with previously learned social behaviours [61]. The immediate family imparts foundational language and role-taking skills, sense of self and interpersonal competence during the early years [61]. Then, through adult socialization dynamics that occur during professional education programming, the individual shifts from individual values and motives to a collectively assimilating socialization process [61].

### 2.3. Bridges’ Transition Theory

Bridges’ transition theory focuses on the subjective psychological processes an individual undergoes during a transition to make meaning of change [62]. Bridges posits that individuals progress through three main phases of transition: endings, the neutral zone and new beginnings. These phases culminate in the achievement of self-development [62]. Endings typically mark the beginning of a transition, a period of letting go and accepting that familiar ways of being have ceased [62]. The difficulty experienced during this phase emanates from the loss of sense of identity, the sudden realization of a new reality, uncertainty about the future and detachment from familiar cues [62]. Separation from the old norms results in liminality [62]. While often marked by emptiness and stagnation, this feeling of being ‘on the margins’ also provides a crucial opportunity for reorientation and rediscovery. Successfully navigating a transition leads to the new beginnings phase, marked by inward resolve to embrace new ways of being, including clear and focused goals [62].

### 2.4. Meleis’ Transition Theory

Meleis’ theory works on the premise that transitions are situation-specific [63,64,65]. Transition is referred to as an internal process of passing from one life phase, condition or status to another [63]. Its occurrence is a result of an individual’s interaction with their environment and is triggered by developmental, situational, organizational or health-related events [63]. Responses to these triggers are influenced by personal factors encompassing the individual’s attached meanings, cultural beliefs and attitudes, socio-economic status, preparations and knowledge about the change, and the prevailing community and societal factors during the change that either facilitate or inhibit their response [63,65]. The theory recognizes the salience of individual and external factors on the outcomes of transition, providing a useful tool for the assessment and development of therapeutics and interventions specific to patients and their families to enhance their transition [63,65]. Clarification of meanings, employing role modelling techniques, specifying targets and debriefing are recommended as essential for fostering and promoting healthy transition behaviours [65].

### 2.5. Schlossberg’s Transition Theory

Schlossberg’s theory focuses on adult life transitions and provides a framework that attempts to explain people’s ability to cope with changes in their lives within the context of individual characteristics and external influences [66]. An occurrence and non-occurrence of life events can both trigger a transition, according to Schlossberg; therefore, a transition is said to have occurred if an event or non-event alters one’s assumptions and worldviews, necessitating a change in behaviour and relationships [66]. The outcome of the transition is dependent on four prominent contextual factors: self, situation, support and strategy. These variables are responsible for idiosyncratic responses to different transitions [66,67]. The self encompasses the mix of strengths and deficits the individual possesses at the transition’s outset and how they can capitalize on their strengths to offset deficits [66,67]. The situation encompasses the distinct features of the transition, such as its timing, duration, nature of onset, degree of stress introduced, familiarity with the change and the quality of support from the family unit, network of friends and community and institutional support [66,67].

### 2.6. Beckhard’s Model for Organizational Change

Beckhard’s model of change response is organization-based and formulated to provide guidelines on how organizations can prepare for an impending change [68]. Beckhard asserts that the interim between the current state and the desired future state represents the transition period that must be managed effectively [68]. Some effective strategies to ensure seamless transition include developing an outline of plans to foster a commitment to change, providing educational and training support, redesigning organizational structures to accommodate ongoing change, creating an awareness of the importance of the change, maintaining open communication lines, identifying critical starting points for the change, assigning roles and responsibilities, clearly defining job descriptions and expectations, and identifying influential individuals to champion the change agenda [68].

### 2.7. Ashforth’s Role Transition in Organizational Life

Ashforth’s theory is identity-based and provides insight into the social-psychological processes that individuals undergo when transitioning from one role to the other. According to Ashforth, in a role transition process, individuals disengage from one role (role exit) and engage with another (role entry) [69]. Individuals often experience ‘liminality’ during the transition, in that they feel somewhat unmoored until they can establish their footing in the new role. As individuals engage in the demands and enactment of the role, they come to feel more authentic as legitimate role-holders [69]. Subsequent incidents that create doubt about longevity in a role can trigger disengagement and a quest for a role more in keeping with expectations [69]. The desire to stay in the new role is sustained by psychological motives [69]. These include a desire for identity, a sense of meaning, belongingness derived from the role and the availability of opportunities for control.

### 2.8. Benner’s Novice to Expert Theory

Benner’s theory is founded on the concepts of the Dreyfus Model of Skill Acquisition [70], which explains how education and experiential learning guide new learners to develop and evolve their skills over time. The Dreyfus model findings were confirmed by Benner in her study of nurses with different levels of experience [33,71]. According to Benner (1984) [33], the process of skill acquisition in nursing progresses through five levels of proficiency: the novice, advanced beginner, competent, proficient and expert levels [33,71]. Student nurses, or nurses entering a clinical area without prior clinical experience in that and, most typically, with no experience in clinical nursing practice, are classified as *novices* [33,71]. Their theoretical knowledge base and limited experience interfere with discretionary judgement as they are unable to connect the general with the particular encountered in clinical situations [33]. They require extensive support, rules and guidelines to navigate demand situations. The advanced beginner comes into a new role with limited clinical experience and associated clinical meaningfulness, resulting in their approach to all tasks being met with the same sense of urgency and priority [33]. Interactions with colleagues and patient engagement are the main learning avenues.

The competent nurse has from one to two years of working experience in a particular practice area and can plan care and predict contingencies for the immediate future because they have accumulated relevant clinical experiences [33]. Over time, the nurse becomes proficient, gaining heightened situational awareness and the ability to recognize notice transitions in patient’s physiological states and responses in unexpected or unfamiliar situations [33]. Finally, the expert nurse, having gained tacit experience and clinical confidence, responds intuitively, fluidly and flexibly to unanticipated clinical demands, utilizing a more perceptual grasp of the whole clinical situation [33].

### 2.9. Spector’s Transition to Practice Regulatory Model

This model was developed in response to a growing need to address NGN transition support challenges as well as safeguard patient safety [72,73]. The model is founded on tenets of preceptorship, experiential learning modules and institutional support [72,73]. Preceptorship forms an integral part of the model, and experiential learning opportunities focused on patient-centred care, communication and teamwork, evidence-based practice, quality improvement and informatics must be provided [72,74].

### 2.10. Kramer’s Reality Shock

Kramer’s reality shock theory was formulated to outline the experiences of new nurses as they transition from nursing school to clinical practice, with the intent of alleviating the stressors experienced during the transition period and offering solutions that promote effective transition, job satisfaction and nurse retention [75]. The emphasis was on resolving the professional-bureaucratic conflicts that alter the new graduate nurse’s vision of the nursing profession and practice [75]. The unexpected and undesirable social, physical and emotional reactions to the startling discovery that the professional values taught during nursing education conflict with work-world values underpin reality shock [75]. The transition from student nurse to professional nurse proceeds through four stages: the honeymoon, shock and rejection, recovery and resolution phases [75]. The honeymoon phase entails initial excitement and desire to make an impact at the professional level [75]. During this period, the NGN is unaware of many of the realities of the nursing profession, and coping ranges from negative feelings toward oneself, colleagues or the workplace or educational institutions that ‘misled’ them, to a total embrace of the new workplace norms with an abandonment of their professional ideals [75]. A new appreciation for the profession’s dynamics develops in the recovery and resolution phases, although encountering new and unfamiliar situations usually results in destabilization or regression [75].

### 2.11. Duchscher’s Transition Theory

Duchscher’s theory builds on Kramer’s work as it captures the entirety of the NGN’s journey into professional practice over the initial 12 months following orientation [32,76]. The concept of transition shock was coined by Duchscher (2009) [76] to represent the process of the initial entry to practice (0–4 months) and its associated experiences of loss, doubt, confusion and disorientation. The new nurse goes through a process of ‘becoming’ a fully-fledged professional while navigating socio-developmental, emotional, intellectual and physical elements that can impact the process [32,38,76]. The transition into a professional role progresses through three stages: doing, being and knowing [32,38,76]. The first 3 to 4 months, known as the doing stage, is a period of awakening prompted by the awareness of disparities between the schoolwork and the clinical work [32,38,76]. A corresponding attempt is made to bridge the existing gaps in knowledge and skills by learning and adopting a task-orientated mentality to fulfill the responsibilities [32,38,76]. Approximately 4 months post-orientation, the NGN progresses into the being stage, experiencing an upward shift in their thought processes, knowledge, skills and confidence level [32,38,76]. Their focus shifts from themselves to the working environment, with inconsistencies revealed within their workplace becoming evident during this period, motivating a search for meaning and reconciliation of their ideals with the realities of the profession [32,38,76]. In the final knowing stage, thinking processes are more future-orientated, and nursing is considered in relation to other professions [32,38,76].

## 3. Theory Application to New Graduate Nurse Transition

Investigations into the NGN’s professional role transition suggest a complex, dynamic and multidimensional period of adjustment to the realities of the nursing profession that is influenced by intrapersonal, interpersonal and organizational factors [32,38,46,76,77,78,79,80,81,82,83,84,85]. The tenets of each transition theory presented here align with these findings and reflect a convergence of situational, individual and collective variables at play during the transition process [61,62,63,66,68,69,76].

The subjective sense-making process illustrated by Bridges (1980) [62] represents the possible inner conflicts and apprehensions of new graduate nurses as they transition into professional roles. The professional role entails increased accountabilities and requires relinquishing the security and privileges associated with the student role. As emphasized by Bridges, the inner turmoil of letting go requires the passage of time for full acceptance of the new normal [62]. Accelerating the process denies people needed closure, interfering with their ability to embrace the change experience [62].

Ashforth (2001) [69] the importance of transition bridges to alleviate the emotional burden of changing roles, as psychological disengagement from the previous role is required for a successful progression. Kramer (1974) [75] offered similar conceptual threads of neophytes seeking to understand how to converge the ideals taught in their schooling with the practicalities of the workplace. This is an example of how implementing strategies such as transition bridges to help them locate the gaps and appreciate the overlaps between education and industry could help graduates mitigate the shock of the discrepancies. Professional role transition is also mediated by individual dispositions. Findings from Baharum et al. (2023) [77] and Feltrin et al. (2019) [86] suggest that new graduates who have forward-thinking personalities, self-confidence and a strong sense of identity adapted more effectively during transitions than those who did not possess these traits. Hardy and Conway (1978) [61] associated such differences in role-transitioning with early childhood socialization, claiming that the family’s subculture is instrumental in personality development and accounts for many of the differences in coping. In addition, modes of adjustment adopted in a new work role must reflect a balance of individual dispositions and organizational factors [59].

Events such as transitioning into professional nursing practice for the first time often prompt an appraisal exercise, involving an evaluation of the self, situation and resources available to help relocate oneself and cope with the perceived change [59,63,66]. Clinical communication deficiencies, the propensity to make mistakes and reliance on experienced colleagues to mitigate the school/practice disconnects undermine their confidence [46,50,87,88]. Unanticipated job demands leave them physically and emotionally exhausted [82,89,90,91,92]. Inadequate preceptoring or mentoring, along with incivility from colleagues, superiors and other members of the care team, compound the already stressful situation [25,93,94,95,96,97,98,99].

Supportive environments are essential to optimizing retention in practice [3,76,79,100,101,102,103,104,105]. Benner (1984) [33], Duchscher (2009) [76] and Kramer (1974) [75] articulated gaps in the neophyte’s knowledge and skills and echoed the need for supportive systems. The neophyte’s primary focus is on meeting role expectations and integrating successfully into their new professional culture [33,36,50,76,101,106,107].Professional integration strategies are crucial in shaping Generation Z’s future intentions regarding the profession [20,41,50,72,74,82,84,108,109,110,111,112]. New professional roles and relationships that fail to satisfy personal expectations and do not evoke a sense of identity and belonging are experienced as more disruptive than convergent and risk alienating the new professional’s sense of autonomy, leading to unnecessary turnover [63,69,72,76].

## 4. Exploring the Needs of the Contemporary New Graduate Nurse

Discrepancies between expected roles, relationships and responsibilities, combined with challenges to knowledge application in new clinical situations of high outcome risk potential, upend the stability, consistency, predictability and familiarity required for a healthy professional adjustment and increase feelings of uncertainty regarding choice of career [38,75,76,83]. Support from a person’s immediate community acts as a critical buffer to transition challenges [63,66,67]. Organizations must be keen on providing an environment that fosters growth and harnesses the strengths of incumbents for both role and personal development [59]. New entrants in most contemporary workforces are of the Generation Z cohort (born between mid-1990 and early 2010) [113,114]. The generational characteristics of this cohort infer the need to design transition-to-practice support programs that consider those characteristics [114,115,116]. According to Beckhard, organizations must continually evaluate their practices and operations to determine if they are effective and comparable to evolving standards [68]. This, Chicca and Shellenbarger (2018) [115] suggest, includes tailored teaching and learning, as well as valuing the voice of contemporary new graduates. Person-specific factors have been identified that can shape and steer behaviour in a transition process [59,61,63,66,67,69]. An individual’s level of awareness, sense of meaning, cultural values, socio-economic status, exposure to similar experiences, interpretation and preparation for anticipated transition experiences all influence their capacity to adapt to a change [57,59,61,62,66,69]. These factors must be assessed at the outset of the transition and routinely thereafter to understand how the individual circumstances of each NGN can inform tailored support and guidance. For instance, study findings from Hussein et al. (2016) [117] revealed that younger NGNs were likely to be more content with their practice environment than older NGNs. This was attributed to family–work conflicts that require older NGNs to balance work and home, coupled with increased personal confidence from prior life and job experience. Increased psychological empowerment, which stems from having a sense of self-efficacy and autonomy, an alignment of one’s values with organizational values and the ability to make an impact within an organization [118] has been associated with increased job satisfaction and retention [119,120]. Richard and Kim (2024) [121] found that the meaningfulness of a job influenced career progression intentions and decisions among early-career nurses.

## 5. A Call to Action

The pressing issues related to the growing trend of global nursing attrition and, in particular, the loss of early to mid-career intellectual capacity and practice experience serve as a threat to the future development of healthy, committed, compassionate and knowledgeable nursing graduates [29,122,123,124]. New nurses should be knowledgeable about the transition experience, gradually introduced to the expectations and demands of their new professional nursing roles and matched with highly trained preceptors and mentors who are afforded manageable work schedules [3,46,95,125,126,127,128,129]. Because the decision to act upon early formative impressions often occurs within the latter part of a transition experience, early interventions are critical [32,108,130,131,132]. Comprehensive and well-structured support that addresses transition challenges has been found to increase confidence in providing care, improve competence, improve clinical leadership skills, increase job satisfaction and lower turnover intentions [57,83,100,111,133,134,135,136].

Ongoing deleterious workplace occurrences such as incivility and bullying interfere with NGNs’ integration and must be addressed by leadership to reflect a commitment to NGNs’ well-being and professional growth [25,84,137,138]. When organizations establish favourable transition strategies and leaders demonstrate authenticity in their leadership, NGNs are more likely to experience job satisfaction and less likely to have turnover intentions [28,128,139,140].

## 6. Conclusions

The transition theories presented here illustrate the multifaceted nature of a new professional’s experience and adaptation process upon entering practice. Nurse leaders, policymakers and stakeholders in health ministries, educational institutions, healthcare organizations and associations can utilize this insight to inform and guide their decisions and actions. Adequately supporting new graduates to transition and integrate into both the healthcare system and their professions is key to building a workforce capable of sustaining the future of the nursing profession and enacting global healthcare plans.
